# Glycyrrhizin Alleviates Neuroinflammation and Memory Deficit Induced by Systemic Lipopolysaccharide Treatment in Mice

**DOI:** 10.3390/molecules181215788

**Published:** 2013-12-17

**Authors:** Jeong-Ho Song, Ju-Won Lee, Beomsoo Shim, Chang-Yeol Lee, Sooyong Choi, Chulhun Kang, Nak-Won Sohn, Jung-Won Shin

**Affiliations:** Department of East-West Medical Science, Graduate School of East-West Medical Science, Kyung Hee University, Yongin 446-701, Korea; E-Mails: beginbegun@hanmail.net (J.-H.S.); won8502@hanmail.net (J.-W.L.); bom-su@hanmail.net (B.S.); cylee81@hanmail.net (C.-Y.L.); csyomd@hanmail.net (S.C.); kangch@khu.ac.kr (C.K.); sohnnw@khu.ac.kr (N.-W.S.)

**Keywords:** glycyrrhizin, neuroinflammation, memory deficit, microglial activation

## Abstract

The present study investigated the effects of glycyrrhizin (GRZ) on neuroinflammation and memory deficit in systemic lipopolysaccharide (LPS)-treated C57BL/6 mice. Varying doses of GRZ was orally administered (10, 30, or 50 mg/kg) once a day for 3 days before the LPS (3 mg/kg) injection. At 24 h after the LPS injection, GRZ significantly reduced TNF-α and IL-1β mRNA at doses of 30 and 50 mg/kg. COX-2 and iNOS protein expressions were significantly reduced by GRZ at doses of 30 and 50 mg/kg. In the Morris water maze test, GRZ (30 mg/kg) significantly prolonged the swimming time spent in the target and peri-target zones. GRZ also significantly increased the target heading and memory score numbers. In the hippocampal tissue, GRZ significantly reduced the up-regulated Iba1 protein expression and the average cell size of Iba1-expressing microglia induced by LPS. The results indicate that GRZ ameliorated the memory deficit induced by systemic LPS treatment and the effect of GRZ was found to be mediated through the inhibition of pro-inflammatory mediators and microglial activation in the brain tissue. This study supports that GRZ may be a putative therapeutic drug on neurodegenerative diseases associated with cognitive deficits and neuroinflammation such as Alzheimer’s disease.

## 1. Introduction

Glycyrrhizin (GRZ), a triterpenoid saponin compound, is the main constituent of *Glycyrrhiza glabra* and is composed of a molecule of glycyrrhetinic acid and two molecules of glucuronic acid [1,2]. GRZ given orally is absorbed into the bloodstream as 18β-glycyrrhetinic acid (GA), then GA reaches the brain through the brain-blood barrier [3]. GRZ has been reported to have various pharmacological effects, including anti-inflammatory and neuroprotective effects. Its anti-inflammatory outcome is by suppressing the expression of pro-inflammatory cytokine genes through the inhibition of nuclear factor-κB (NF-κB) and phosphoinositide-3-kinase (PI3K) activity [4], and by attenuating excessive nitric oxide (NO) and reactive oxygen species (ROS) production [4,5]. GRZ also suppresses inducible nitric oxide synthase (iNOS) expression and reduces prostaglandin E2 (PGE2) release through the inhibition of cyclooxygenase-2 (COX-2) [6,7]. It is suggested that the inhibitory action of GRZ on NF-κB and PI3K activities protect neurons from glutamate-induced excitotoxicity and ischemic injury [8,9]. Recently, a line of *in vivo* studies reported that GRZ exerted neuroprotective effects against cerebral ischemia, intracerebral hemorrhage, and ischemic spinal cord injury via its anti-inflammatory effects [10,11,12,13]. These reports suggested that GRZ plays an inhibitory role on high mobility group box 1 (HMGB1) protein. HMGB1 behaves like an early pro-inflammatory cytokine to promote inflammation [14] and serves as a risk factor for memory impairment, neurodegeneration, and progression of neuroinflammation [15]. Moreover, previous reports exhibited that GRZ has spatial memory enhancing effect [1] and ameliorating effect on cognitive impairment induced by beta-amyloid (Aβ) injection into the hippocampus [16]. The cognitive ameliorating effect of GRZ was supported by its suppressing effect on Aβ-induced microglial activation and inflammation *in vitro* and *in vivo* [17].

Neuroinflammation, the inflammation associated with the brain, is characterized by the activation of microglia and expression of major inflammatory mediators without typical features of peripheral inflammation such as edema and neutrophil infiltration [18]. Neuroinflammation causes cognitive impairment, even if it is acutely stimulated by immunostimulatory component such as lipopolysaccharide (LPS) [19]; and its chronic state contributes to progression of neurodegenerative diseases including Alzheimer’s disease (AD) [20]. Systemic treatment of LPS stimulates the inflammatory responses in the brain through the toll-like receptor-4 mediated signaling pathway [21]. Upon exposure to LPS, microglia are activated and produce pro-inflammatory mediators such as cytokines, chemokines, prostanoids, and reactive oxygen species [22]. Microglia are the primary cellular source of pro-inflammatory cytokines, including tumor necrosis factor-α (TNF-α) and interleukin (IL)-1β, detected in the brain [23]. Pro-inflammatory cytokines disrupt hippocampal neuronal functions such as long-term potentiation (LTP) and working memory consolidation [24,25]. Consequently, LPS induces a complex array of behaviors known as “sickness behaviors” [26] and leads to alterations in central processes involved in learning and memory [19,27]. Therefore, systemic or intraventricular LPS injection into rodents is popularly used as a model for studying the interaction between inflammation, brain functions, and memory deficits [19,24,25,27].

To better understand anti-neuroinflammatory effect of GRZ, the present study investigated its effects on TNF-α, IL-1β, COX-2, and iNOS expression in the brain tissue; microglial activation in the hippocampus; and on learning and memory deficits induced by systemic LPS treatment in mice.

## 2. Results and Discussion

### 2.1. Effects on TNF-α and IL-1β Expressions in the Brain Tissue of LPS-Treated Mice

**Figure 1:**
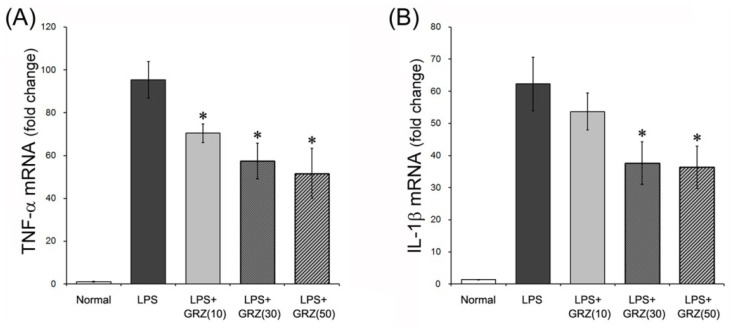
Effects of GRZ on TNF-α and IL-1β mRNA in the brain tissue. GRZ significantly attenuated the up-regulation of brain TNF-α mRNA at all doses of 10, 30 and 50 mg/kg (**A**) IL-1β mRNA was attenuated at 30 and 50 mg/kg of GRZ; and (**B**) Data are represented by mean ± SEM (n = 6 in each group; * *p* < 0.05 compared to the LPS group).

**Figure 2:**
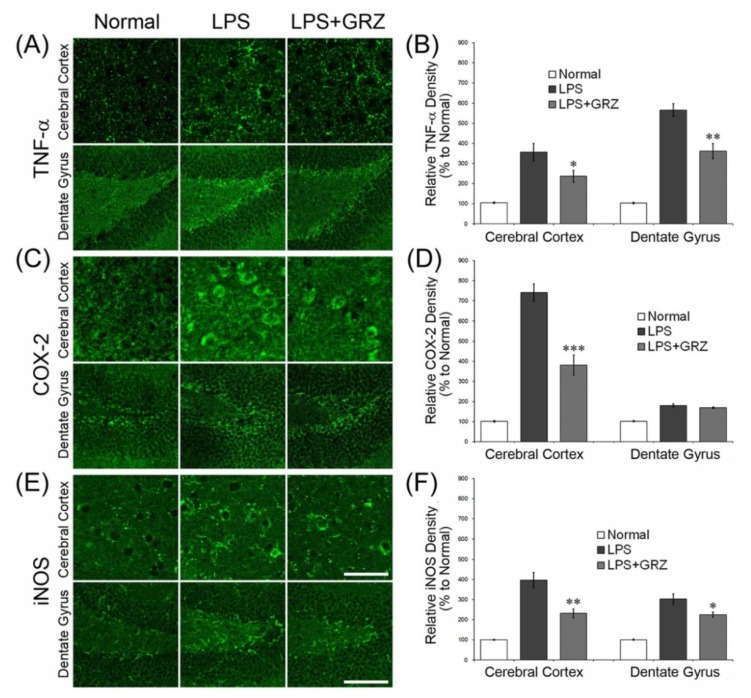
Effect of GRZ on TNF-α, COX-2, and iNOS expression in brain tissue immunohistochemistry. Representative TNF-α-, COX-2- and iNOS-expressing cells in the brain tissue of mice which performed the Morris water maze test. Scale bars are 100 μm (**A**,**C**,**E**) GRZ (30 mg/kg) significantly attenuated the relative immune-densities of TNF-α **(B)** COX-2 (**D**) and iNOS; and (**F**) in the cerebral cortex or in the dentate gyrus of hippocampus. Data are represented by mean ± SEM (n = 6 in each group; * *p* < 0.05; ** *p* < 0.01; *** *p* < 0.001 compared to the LPS group).

Systemic treatment of LPS stimulates pro-inflammatory cytokines, including TNF-α and IL-1β, in the brain. Compared to serum, the brain showed significantly higher levels of TNF-α and IL-1β, 28 h post a single systemic injection of LPS [28]. Additionally, mRNA/protein expression of inflammatory mediators in the brain appeared within 4–8 h and subsided in 1–3 days after a single LPS injection [29,30]. In this study, TNF-α and IL-1β mRNA levels were measured 24 h after LPS (3 mg/kg) injection. LPS induced robust increases of TNF-α and IL-1β mRNA in the brain tissue compared to the normal group. GRZ treatment significantly reduced TNF-α mRNA level at all doses of 10, 30 and 50 mg/kg (*p* < 0.05; respectively) and decreased IL-1β mRNA level at doses of 30 and 50 mg/kg (*p* < 0.05; respectively) compared to the LPS group (Figure 1). In addition, TNF-α expression was observed with immunohistochemistry in the brain tissue of the mice which performed the Morris water maze test. The LPS group showed significant increases of relative density of TNF-α expression in the cerebral cortex (*p* < 0.001) and dentate gyrus (DG) region of the hippocampus (*p* < 0.001) compared to that of the normal group. The LPS+GRZ group (30 mg/kg) demonstrated significant reductions in relative densities of TNF-α expression in the cerebral cortex (*p* < 0.05) and DG region of the hippocampus (*p* < 0.01) compared to that of the LPS group (Figure 2A,B). The results in this study indicate that GRZ reduced the over-expression of TNF-α and IL-1β induced by systemic LPS treatment in the brain tissue. Previous reports have demonstrated that GRZ effectively suppressed TNF-α and IL-1β expression in ulcerative colitis [31], in post ischemic brain [10], and in spinal cord injuries [12,13]. The results suggest that GRZ exerts anti-neuroinflammatory effects.

### 2.2. Effects on COX-2 and iNOS Expressions in the Brain Tissue of LPS-Treated Mice

**Figure 3:**
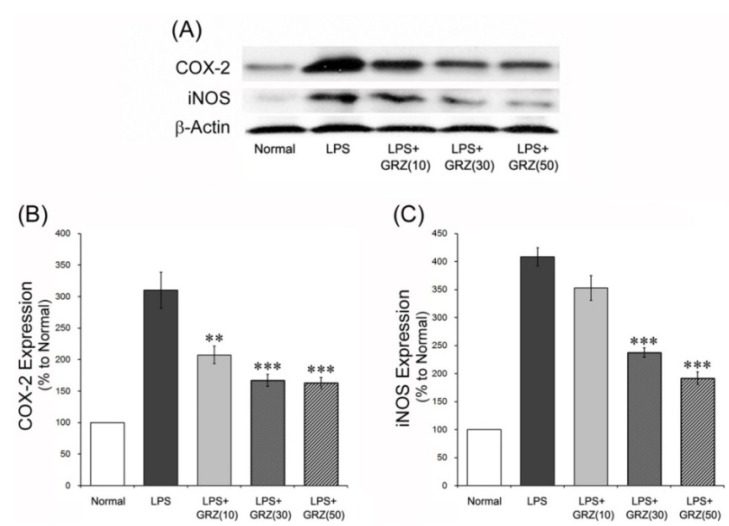
Effects of GRZ on COX-2 and iNOS protein expression in the brain tissue. Representative western blots illustrating differences in the bands of COX-2 and iNOS (**A**) GRZ significantly attenuated the up-regulation of brain COX-2 expression at all doses of 10, 30 and 50 mg/kg; (**B**) and iNOS expression was attenuated at 30 and 50 mg/kg of GRZ; and (**C**) Data are represented by mean ± SEM (n = 6 in each group; ** *p* < 0.01; *** *p* < 0.001 compared to the LPS group).

COX-2 plays a central role in the inflammatory cascade by producing prostaglandin in acute and chronic inflammatory conditions [32]. NO is a major pleiotrophic mediator produced by iNOS and reacts with superoxide to form the powerful oxidant peroxynitrite [33]. In this study, COX-2 and iNOS expressions were measured 24 h post LPS injection using a western blotting method. GRZ treatment significantly reduced the increase of COX-2 expression at all doses of 10, 30 and 50 mg/kg (*p* < 0.01, *p* < 0.001, *p* < 0.001; respectively) and also reduced the increase of iNOS expression at doses of 30 and 50 mg/kg (*p* < 0.05, *p* < 0.01; respectively) compared to the LPS group (Figure 3). In addition, COX-2 and iNOS expressions were observed with immunohistochemistry in the brain tissue of the mice which performed the Morris water maze test. The LPS+GRZ group (30 mg/kg) demonstrated significant reductions in relative densities of COX-2 and iNOS expression in the cerebral cortex and DG region of the hippocampus compared to that of the LPS group (Figure 2C–F). The results support that GRZ exerts anti-neuroinflammatory effect, considering a line of results in previous studies and its effects on TNF-α and IL-1β mRNA in the present study. GRZ has been shown to suppress COX-2 and iNOS expressions in acute lung injury induced by LPS treatment [6]. GRZ also showed an anti-inflammatory effect by attenuating the generation of excessive NO, PGE2, and ROS through the inhibition of NF-κB and PI3K activity in various *in vitro* and *in vivo* studies [4,5,6,7,8].

### 2.3. Effect on Microglial Activation in the Hippocampal Tissue of LPS-Treated Mice

**Figure 4:**
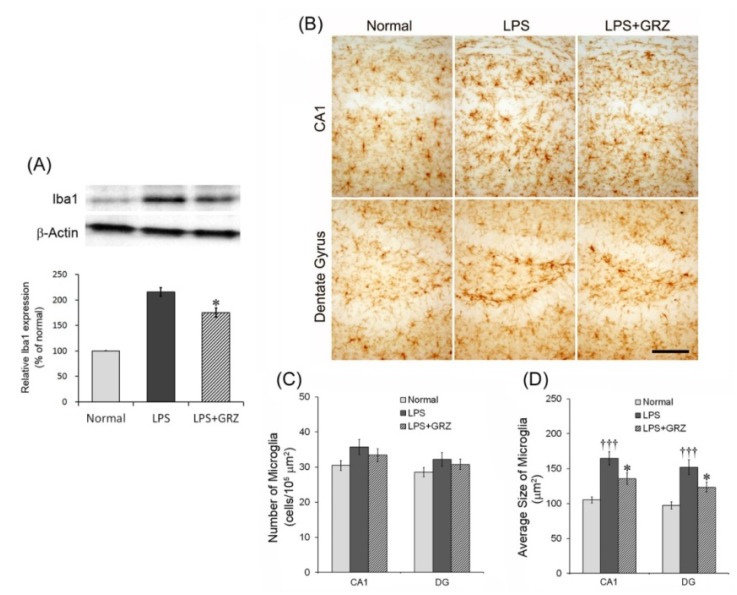
Effects of GRZ on Iba1 protein expression and Iba1-expressing microglia in the hippocampal tissue. Representative western blots illustrating differences in the bands of Iba1 (**A**) GRZ (30 mg/kg) significantly attenuated the up-regulation of hippocampal Iba1 expression of mice which performed the Morris water maze test. Representative Iba1-expressing microglia in the hippocampus of mice which performed the Morris water maze test. Scale bars are 100 μm (**B**) The numbers of Iba1-expressing microglia were not different between groups (**C**). The average cell size of Iba1-expressing microglia was significantly attenuated at 30 mg/kg of GRZ; and (**D**) Data are represented by mean ± SEM (n = 6 in each group; ††† *p* < 0.001 compared to the normal group; * *p* < 0.05 compared to the LPS group).

To better understand GRZ’s anti-neuroinflammatory effect in the hippocampus, observation of microglial activation in the hippocampal tissue of mice was performed in the Morris water maze test. Microglia are key cellular elements of the acute neuroinflammatory response and the primary source for pro-inflammatory cytokines detected in the brain [23]. Systemic LPS injection promotes neuroinflammation through microgial activation and overproduction of inflammatory cytokines [34]. Activated microglia are also responsible for the induction of COX-2 and iNOS in the brain following LPS stimulation [35]. Ionized calcium binding adaptor molecule 1 (Iba1) protein expression, a marker of microglial activation in the hippocampal tissue was measured 24 h after the LPS injection using Western blotting method. GRZ treatment significantly reduced Iba1 expression at a dose of 30 mg/kg (*p* < 0.05) compared to the LPS group (Figure 4A). When microglia are activated in response to immunological stimulation such as LPS, they undergo morphological changes which include shortening and thickening of processes and cell size increase [23]. Therefore, cell number and cell size of Iba1-expressing microglia in the CA1 and DG region of the hippocampus were measured. Although GRZ treatment did not attenuate the number of Iba1-expressing microglia both in the CA1 and DG region of the hippocampus compared to that of the LPS group (Figure 4B,C), the average cell size of the Iba1-expressing microglia was significantly decreased by GRZ treatment both in the CA1 (*p* < 0.05) and DG region (*p* < 0.05) of the hippocampus compared to that of the LPS group (Figure 4B,D). The results indicate that GRZ attenuated the microglial activation related over-expression of pro-inflammatory cytokines in the hippocampus induced by systemic LPS treatment.

### 2.4. Effect on Spatial Learning of LPS-Treated Mice

**Figure 5:**
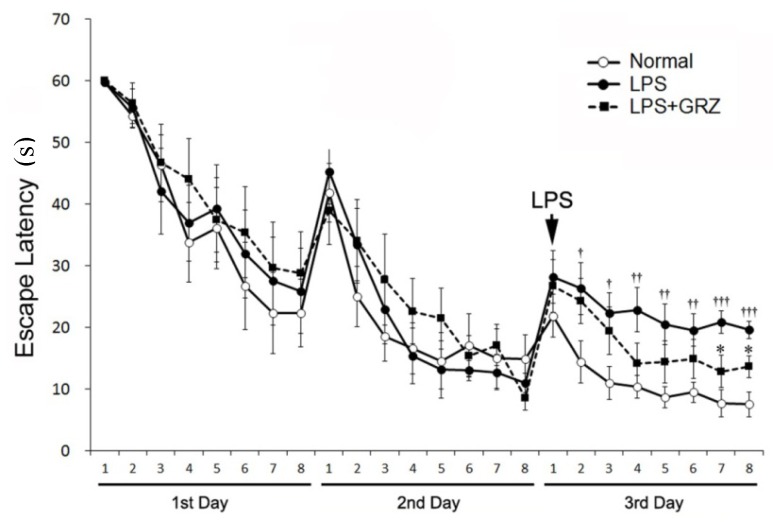
Effect of GRZ on the acquisition training trials. The escape latencies on the 1st and 2nd day were not different among the normal, LPS, and LPS+GRZ groups (F_2,21_ = 0.33, *p* = 0.723; F_2,21_ = 0.13, *p* = 0.882; respectively). The LPS + GRZ group showed significantly shorter escape latency at the 7th and 8th trials on the 3rd day, while the escape latency for the total trials on the 3rd day was significantly different from the LPS group (F_1,14_ = 1.98, *p* = 0.181). Data are represented by mean ± SEM (n = 12 in each group; † *p* < 0.05; †† *p* < 0.01; ††† *p* < 0.001 compared to the normal group; * *p* < 0.05 compared to the LPS group).

TNF-α and IL-1β are the most potent pro-inflammatory cytokines to induce behavioral alterations. Elevated levels of TNF-α have been demonstrated in AD patients [36]. TNF-α protein synthesis inhibitor reversed cognitive deficits induced by chronic LPS-infusion into the ventricle of rats [37]. IL-1β reduced adult hippocampal neurogenesis [38], induced synaptic loss of hippocampal neurons [39], and aggravated long-term potentiation (LTP) and synaptic plasticity in the hippocampus [40]. Direct central administration of IL-1β impaired hippocampal-dependent learning and memory [41]. Moreover, microglial activation is involved in learning and memory impairment through the release of TNF-α and IL-1β and the negative impact on hippocampal LTP [42]. There are numerous reports that systemic LPS treatment produces learning and memory impairment, even if it is acutely stimulated by a single injection of LPS [19]. In the acquisition trials before the LPS treatment, all study groups showed relatively comparable results in the escape latency on the 1st day (F_2,21_ = 0.255, *p* = 0.777) and 2nd day (F_2,21_ = 0.159, *p* = 0.854). After LPS treatment on the 3rd day, the escape latency of the LPS group was significantly longer compared to the normal group (F_1,14_ = 30.16, *p* < 0.001), while the escape latency between groups (normal, LPS, and LPS+GRZ) was significantly different (F_2,21_ = 12.19, *p* < 0.001). The LPS + GRZ group showed significantly shorter escape latency at the 7th and 8th trials on the 3rd day (*p* < 0.05, respectively), while the escape latencies for all the trials on the 3rd day were significantly shorter than that of the LPS group (F_1,14_ = 5.164, *p* < 0.05) (Figure 5). The results indicate that pre-treatment of GRZ was effective in improving spatial learning of LPS-treated mice.

### 2.5. Effects on Memory Deficit of LPS-Treated Mice

**Figure 6:**
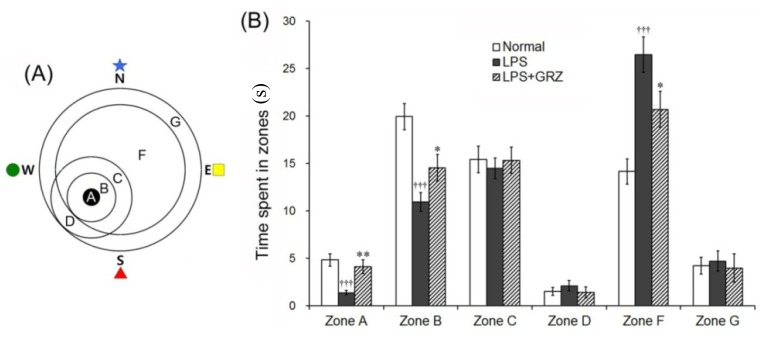
Effects of GRZ on the swimming time spent in discrete zones. Computerized grid design used in the retention test. Discrete zones are labeled with letters, zone A representing the platform site (**A**) GRZ significantly prolonged the swimming time spent in zones A and B, while significantly shortened in zone F; and (**B**) Data are represented by mean ± SEM (n = 12 in each group; ††† *p* < 0.001 compared to the normal group; * *p* < 0.05; ** *p* < 0.01 compared to the LPS group).

**Figure 7:**
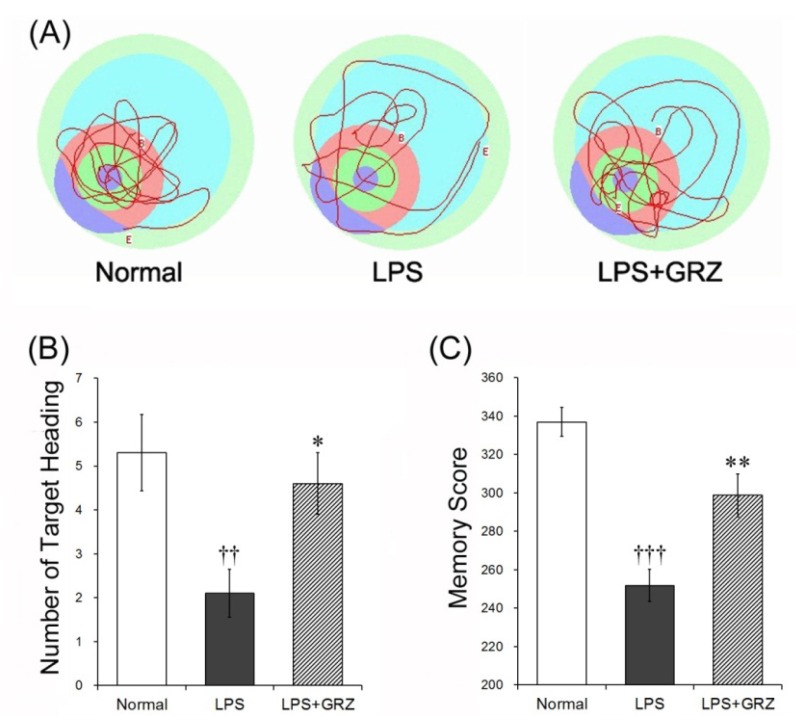
Effects of GRZ on the retention memory test. Representative swimming tracts of the normal, LPS, and LPS+GRZ groups (**A**) GRZ significantly increased the number of target heading on platform site (**B**) and memory score; and (**C**) in the retention test. Data are represented by mean ± SEM (n = 12 in each group; †† *p* < 0.01; ††† *p* < 0.001 compared to the normal group; * *p* < 0.05; ** *p* < 0.01 compared to the LPS group).

In the retention trial on the 4th day of the Morris water maze test, the swimming time spent in the various zones, number of the target heading and memory score were analyzed with a grid design of six zones (Figure 6A) and were used to estimate spatial memory. The LPS group spent significantly less time in zones A (the target, *p* < 0.001) and B (peri-target area, *p* < 0.001), but significantly more time in zone F (dis-target area, *p* < 0.001) compared to those of the normal group. GRZ treatment significantly prolonged the swimming time spent in zones A (*p* < 0.01) and B (*p* < 0.01) and significantly shortened the swimming time spent in zone F (*p* < 0.05) compared to that of the LPS group (Figure 6B). The number of target heading in the LPS group was significantly reduced (*p* < 0.01) compared to the normal group, while GRZ treatment significantly increased the number of target heading (*p* < 0.05) compared to the LPS group (Figure 7A,B). The LPS group revealed significantly lower memory score (*p* < 0.001) than that of the normal group, while the memory score of the LPS + GRZ group was significantly higher (*p* < 0.01) than that of the LPS group (Figure 7A,C). The results indicate that pre-treatment of GRZ was effective in ameliorating spatial memory of LPS-treated mice. The average swimming speed (cm/sec) in the retention trials was not different among three groups (normal, 21.9 ± 1.5; LPS, 20.2 ± 0.5; LPS + GRZ, 21.3 ± 1.2). The result suggests that GRZ might not affect locomotor and emotional behavior.

Recently, Zhu *et al.* and Zhao *et al.* demonstrated that diammonium glycyrrhizinate, a salt form of GRZ, significantly decreased the escape latency and search distance and increased the target crossing times of Aβ(1-42)-induced AD mice [16,17]. The previous reports showed the anti-inflammatory effect of GRZ using *in vitro* BV-2 cells. This study demonstrated the anti-neuroinflammatory effect of GRZ using the brain tissue *in vivo* and used a salt form of GRZ, monoammonium glycyrrhizinate, which has more stable and more significant bioactivities than GRZ. TNF-α and IL-1β inhibit LTP in the CA1 and the dentate gyrus regions of the hippocampus [43]. COX-2 inhibition improves suppression of memory and synaptic plasticity [44]. iNOS upregulation interrupts memory consolidation by altering cholinergic function [45]. Therefore, suppression of these neuroinflammatory mediators can lead to improvement in cognitive function. In the present study, GRZ significantly reduced the up-regulations of TNF-α, IL-1β, COX-2, and iNOS in the brain tissue induced by LPS treatment. Considering all results in this study, it is suggested that GRZ effectively ameliorated the memory deficits induced by systemic LPS treatment through the inhibition of pro-inflammatory cytokines and microglial activation in the brain tissue.

## 3. Experimental

### 3.1. Animals

Male C57BL/6 mice (25–28 g, Nara Biotechnology, Seoul, Korea) were used for this study. All animal protocols were approved by the Ethics Committee for the Care and Use of Laboratory Animals at Kyung Hee University. The animals were housed in plastic cages at constant temperature (22 ± 2 °C) and humidity (55 ± 10%) with 12 h–12 h light-dark conditions. The animals were allowed free access to food and water before the experiment.

### 3.2. Materials

**Figure 8:**
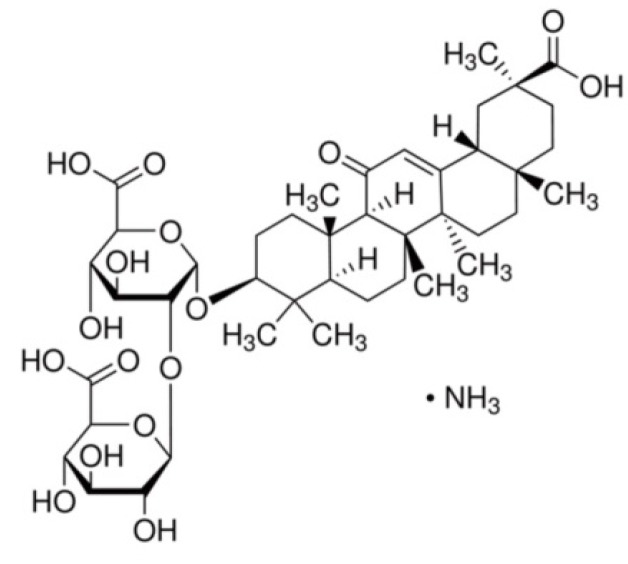
Chemical structure of GRZ (a salt form, monoammonium glycyrrhizinate).

Glycyrrhizin (a salt form of GRZ; monoammonium glycyrrhizinate from *glycyrrhiza* root, C_42_H_62_O_16_·NH_3_; Figure 8) and lipopolysaccharide (LPS from *Escherichia coli* O55:B5) were purchased from Sigma-Aldrich (St. Louis, MO, USA). Anti-COX-2 antibody was purchased from Cayman Chemical (Ann Arbor, MI, USA); Anti-iNOS) antibody from BD Biosciences (Laguna Hills, CA, USA); Anti-Iba1 antibodies (#016-20001, #019-19741) from Wako Pure Chemical Industries (Osaka, Japan); Anti-β-actin antibody from Chemicon International (Temecula, CA, USA); Anti-TNF-α antibody from Santa Cruz Biotechnology (Santa Cruz, CA, USA); and Cy2-conjugated donkey anti-mouse or donkey anti-rabbit IgG from Jackson ImmunoReseach Laboratories (West Grove, PA, USA).

### 3.3. Experimental Groups

For the quantitative real-time polymerase chain reaction (PCR) and western blotting studies, mice were randomly divided into five groups. The normal group (Normal) was allowed free access to food and water without any treatment. The control group (LPS) was intraperitoneally (i.p.) injected with a single dose of LPS (3 mg/kg) and received vehicle (normal saline) orally. The GRZ treatment groups [LPS + GRZ(10), LPS + GRZ(30), LPS + GRZ(50)] were administered GRZ (10, 30, or 50 mg/kg, dissolved in normal saline, orally) respectively, once daily for 3 days before receiving LPS injection. For the Morris water maze study, mice were randomly divided into three groups. The normal group (Normal) was also allowed free access to food and water without any treatment. While the control group (LPS) was intraperitoneally injected with a single dose of LPS (3 mg/kg) on the 3rd day of the experiment. The GRZ treatment group (LPS + GRZ) was administered 30 mg/kg of GRZ, a reliable dose from the PCR and western blotting studies, once daily for 3 days prior to the LPS injection. They were also administered one additional dose 2 h before the retention test on 4th day of the experiment. A total of 66 mice, 30 mice for the PCR and the western blotting study and 36 mice for the Morris water maze study were used.

### 3.4. Real-Time PCR Measurement

Pro-inflammatory cytokines, TNF-α and IL-1β in the brain tissue were measured using real-time PCR method. Twenty-four hours after the LPS injection, the mice were sacrificed by decapitation and the brain was rapidly dissected on ice. Total RNA was extracted from the samples with Trizol (Qiagen, Germany) according to the manufacturer’s protocol. One microgram of total RNA was transcribed into DNA using iScript cDNA synthesis Kit (Bio-Rad, Hercules, CA, USA). After reverse transcription, quantitative real-time PCR was performed using preoptimized primer/probe mixture with iQ SYBR Green Supermix kit (Bio-Rad) and the CFX 96 RT-PCR Detection System (Bio-Rad). Primer sequences for the analyzed genes were as follows: (1) TNF-α; forward, 5'-TGA GAA GTT CCC AAA TGG C-3'; reverse, 5'-GCT ACA GGC TTG TCA CTC-3'; (2) IL-1β; forward, 5'-TGA GCA CCT TCT TTT CCT TCA-3'; reverse, 5'-TTG TCT AAT GGG AAC GTC ACA C-3'; (3) β-actin; forward, 5'-TTT CCA GCC TTC CTT GGG TAT G-3'; reverse, 5'-CAC TGT GTT GGC ATA GAG GTC TTT AC-3'. The relative difference in expression between samples is represented by cycle time values normalized to the measurement of the housekeeping gene β-actin as a reference. The sample values represent x-fold differences from a normal sample (given as a designated value of 1) within the same experiment.

### 3.5. Western Blotting

COX-2 and iNOS expression in the brain tissue and Iba1 expression in the hippocampal tissue were measured by Western blotting method. The brain tissue was homogenized and sonicated on ice in lysis buffer (50 mM Tris–HCl, pH 8.0, 150 mM NaCl, 1% Triton X-100, 0.5% sodium deoxycholate, 0.1% sodium dodecyl sulfate (SDS), 1 mM EDTA, 1% protease inhibitor cocktail; Sigma). After centrifugation, the supernatant was collected and assayed for protein concentration using the Bradford method. Lysate samples containing 50 μg of protein were fractionated by SDS—10% polyacrylamide gel electrophoresis, and then subjected to western blot analysis. The primary antibodies used in this study were mouse anti-COX-2 antibody (#160106, Cayman), mouse anti-iNOS antibody (#610329, BD Biosciences), rabbit anti-Iba1 antibody (#016-20001, Wako) and mouse anti-β-actin antibody (Chemicon). Iba1 expression in the hippocampal tissue was examined in the mice which performed the Morris water maze test.

### 3.6. Morris Water Maze Test

The Morris water maze test was performed for 4 days. The acquisition training was performed for 3 days prior to the LPS injection and the retention test on the 4th day. The apparatus consisted of a circular water pool 100 cm in diameter and 40 cm in height. It was filled with 23 ± 1 °C water with a depth of 28 cm and covered a black platform (10 cm in diameter). The platform was submerged approximately 0.5 cm below the surface of water. The pool was divided into four quadrants: northeast (NE), northwest (NW), southeast (SE), and southwest (SW) at equal distances on the rim. The platform was located in the center of the southwest quadrant. During the first 3 days of acquisition tests, mice were given eight trials per day to find the hidden platform. Each mouse (12 mice per group) was gently placed into the water facing the wall in the direction of north (N), east (E), south (S), and west (W) in two series of order. The mouse was allowed to swim until they reached the hidden platform (maximum swim time was 60 s). The escape latency to reach the platform was recorded and they remained on the platform for 20 s before being removed. The mouse which failed to find the platform within 60 s was guided to the hidden platform and then was placed on the platform for 20 s for reinforcement before being removed. On the 3rd day, LPS was injected into the mice 1 h before the acquisition test.

One trial of the retention test without the platform was performed on the 4th day, 24 h after the LPS injection, to assess the memory of the correct platform location. The mice were placed into the pool and swam freely for 60 s. The swimming paths were recorded by a video camera linked to a computer-based image analyzer (SMART 2.5 video-tracking system, Panlab, Barcelona, Spain). The number of target heading and the swimming time in each zone was analyzed with a grid design of 6 zones (Figure 6A). This grid design, constructed with a computer-based image analyzer, was superimposed over the maze and viewed on a monitor. Memory scores were calculated using the formula (time in zone A × 10) + (time in zone B × 8) + (time in zone C × 6) + (time in zone D × 3) + (time in zone F × 2) + (time in zone G × 1) = memory score. The grid design and the formula for calculating the memory score were based on and modified from the behavior study of Smith *et al.* [46]. The mice were sacrificed after the retention test trial and the brains were randomly used either for Iba1 western blotting (6 mice) or immunohistochemistry (6 mice).

### 3.7. Immunohistochemistry

After the retention test trail, the mice were sacrificed and the brains (6 mice per group, randomly) were used for immunohistochemistry against TNF-α, COX-2, and iNOS expression in the brain tissue. The mice were deeply anesthetized and perfused transcardially with 0.05 M phosphate-buffered saline (PBS) containing 4% paraformaldehyde. The brain was removed and was postfixed in the same perfusing solution overnight at 4 °C. Thirty μm thick coronal sections of brain tissue were made using a freezing microtome (Leica, Wetzler, Germany). The brain sections were rinsed with 0.05 M PBS and incubated for 15 min in 1% hydrogen peroxide PBS at room temperature. The sections were incubated overnight at 4 °C with primary antibodies against TNF-α (1:200, sc-1349, Santa Cruz), COX-2 (1:200, #160106, Cayman), and iNOS (1:200, #610329, BD), then incubated with anti-rabbit or anti-mouse Cy2 as a secondary antibody (Jackson ImmunoResearch, West Grove, PA, USA). The fluorescence-labeled images were captured using a confocal laser-scanning microscope (Carl Zeiss, LSM 510 META, Oberkochen, Germany). The fluorescence-labeled densities against TNF-α, COX-2, and iNOS were analyzed using ImageJ software (Ver. 1.44p, NIH, Bethesda, MD, USA) in corresponding areas. The sample values represent the percentage increase differences from a sample of the normal group and the mean values for the four sections in each mouse were used for statistical analysis. For Iba1 immunohistochemistry, Iba1 (1:500, #019-19741, Wako) was used for primary antibody and the avidin–biotin complex (Vector Laboratories, Burlingame, CA, USA) method were carried out with peroxidase coupling in a mixture containing 0.05% diaminobenzidine (Sigma-Aldrich) and 0.03% H2O2 for 2–5 min. The Iba1-expressing microglia were captured using a light microscope (BX51, Olympus, Tokyo, Japan) equipped with CCD camera (DP70, Olympus) and analyzed using the ImageJ software (Ver. 1.44p, NIH, USA). The number and the average size of microglia in the CA1 and DG region of the hippocampus were measured on an inverted black-white binary image by determination of threshold gray value and pixels definition using the ImageJ software. Data were normalized with the same area (105 μm2) and the mean values from the four sections analyzed in each mouse were used for statistical analysis.

### 3.8. Statistical Analysis

Study data are presented as means ± standard errors. Differences between groups were evaluated using paired Student’s t-test and one-way Analysis of variance (ANOVA). A probability value of less than 0.05 was used to indicate a significant difference.

## 4. Conclusions

This study demonstrates that GRZ effectively reduced neuroinflammation and ameliorated the memory deficits induced by systemic LPS treatment. The effects of GRZ were found to be mediated through the inhibition of pro-inflammatory cytokines and microglial activation in the brain tissue. Therefore, this study supports that GRZ may be a putative therapeutic drug on neurodegenerative diseases associated with cognitive deficits and neuroinflammation such as AD.
